# Poly(Trimethylene Carbonate-co-ε-Caprolactone) Promotes Axonal Growth

**DOI:** 10.1371/journal.pone.0088593

**Published:** 2014-02-27

**Authors:** Daniela Nogueira Rocha, Pedro Brites, Carlos Fonseca, Ana Paula Pêgo

**Affiliations:** 1 INEB – Instituto de Engenharia Biomédica, Universidade do Porto, Porto, Portugal; 2 FEUP - Faculdade de Engenharia da Universidade do Porto, Porto, Portugal; 3 Nerve Regeneration Group, IBMC – Instituto de Biologia Molecular e Celular, Universidade do Porto, Porto, Portugal; 4 ICBAS – Instituto de Ciências Biomédicas Abel Salazar, Universidade do Porto, Porto, Portugal; Politecnico di Milano, Italy

## Abstract

Mammalian central nervous system (CNS) neurons do not regenerate after injury due to the inhibitory environment formed by the glial scar, largely constituted by myelin debris. The use of biomaterials to bridge the lesion area and the creation of an environment favoring axonal regeneration is an appealing approach, currently under investigation. This work aimed at assessing the suitability of three candidate polymers – poly(ε-caprolactone), poly(trimethylene carbonate-co-ε-caprolactone) (P(TMC-CL)) (11∶89 mol%) and poly(trimethylene carbonate) - with the final goal of using these materials in the development of conduits to promote spinal cord regeneration. Poly(L-lysine) (PLL) coated polymeric films were tested for neuronal cell adhesion and neurite outgrowth. At similar PLL film area coverage conditions, neuronal polarization and axonal elongation was significantly higher on P(TMC-CL) films. Furthermore, cortical neurons cultured on P(TMC-CL) were able to extend neurites even when seeded onto myelin. This effect was found to be mediated by the glycogen synthase kinase 3β (GSK3β) signaling pathway with impact on the collapsin response mediator protein 4 (CRMP4), suggesting that besides surface topography, nanomechanical properties were implicated in this process. The obtained results indicate P(TMC-CL) as a promising material for CNS regenerative applications as it promotes axonal growth, overcoming myelin inhibition.

## Introduction

When an injury is inflicted to the spinal cord, the blood-brain barrier (BBB) breaks down locally and a massive infiltration of immune cells is observed. After the initial mechanical trauma (primary damage), cell damage is triggered such that within hours the injury site and the surrounding haemorrhagic areas begin to undergo necrosis (secondary damage), a progressive process that can last for several days. As the necrotic tissue is removed by macrophages, large fluid-filled cavities develop, which are bordered by areas of glial/connective tissue scarring. Even though this glial scar may provide several beneficial functions such as the restoration of the BBB, prevention of a devastating inflammatory response and limit the action of cellular degeneration [1], [2], it also contributes to the establishment of a physical and chemical barrier to axonal regeneration [1]. Strategies aimed at preventing primary and delaying secondary damage need to be administered within minutes to hours after injury making these unsuitable for the spinal cord injury (SCI) patients in a chronic stage [3]. Furthermore, none of the clinical approaches currently available to control or minimize the impact of a SCI lead to neuronal regeneration [4], nor there is an efficient regenerative therapeutic strategy for SCI treatment [4]. Although injured axons show the ability to regenerate when in a peripheral nervous system environment [5], the major factor contributing to the failure of the central nervous system (CNS) regeneration is the lack of capacity of injured axons to spontaneously regenerate in the glial scar microenvironment [6].

The use of biocompatible biomaterials to bypass the glial scar is one of the promising approaches being investigated to promote spinal cord regeneration [3], [7], [8], [9], [10], [11], [12], [13]. These tissue-engineering approaches are usually based on the use of either cell-free bridges or of cellularized biomaterial-based matrices. There are some advantages in the use of a cell-free bridging material, as on one hand cell purification and expansion methods are laborious, time consuming and expensive, and on the other hand when the transplantation of allogenic cells is required, the use of immunosuppressants cannot be circumvented [13]. Therefore, the idea of a cell-free bridging material that uses and controls endogenous cell population responses by having the ability to promote axon regeneration and control inflammatory and glial reactions is arguably appealing.

There are numerous polymeric materials under study for application in nerve repair strategies [3], [10], [14]. These can simultaneously provide a scaffold for tissue regeneration, serve as a cell-delivery vehicle and a reservoir for sustained drug delivery [15]. Within this class of materials, biodegradable polymers are particularly advantageous for the preparation of these bridges, as polymer degradation can be tuned to match the neuronal cell growth. Besides the degradation rate, the mechanical properties of the selected material are also of extreme relevance and a property that can be fitted to one needs. While the implantable structures must be flexible but relatively strong, as well as easy to handle by surgeons, their mechanical properties have an influence on cell phenotype as well [16], [17], [18], [19].

Poly(trimethylene carbonate-co-ε-caprolactone) (P(TMC–CL)) copolymers with high caprolactone (CL) content or the parental trimethylene carbonate (TMC) homopolymer are very flexible and tough materials that can be processed into highly porous three dimensional structures with degradation rates that can be modulated by adjusting the co-monomer content [20], [21]. As P(TMC-CL) has been shown to be processable in a variety of shapes and forms, including porous conduits [22] and electrospun fibers [23], it presents itself as a valuable tool in the design of new strategies for application in the treatment of spinal cord lesions. These materials have been shown to be biocompatible [21], [24] and have been previously explored for peripheral nerve regeneration conduits [20], [22], [24], [25]. Additionally, polymer degradation occurred with minimum swelling of the material [24], which is also an essential feature to prevent nerve compression that could compromise regeneration.

After the promising results obtained in the context of peripheral nerve regeneration, the suitability of P(TMC-CL) copolymers for application in the CNS and the possibility to modulate the biological response by tuning the surface properties at the nanoscale was explored, with the ultimate goal of contributing to the design of an artificial 3D scaffold able to promote spinal cord regeneration. Films based on a P(TMC-CL) copolymer with a high CL content and the respective homopolymers were prepared and cortical neuron cultures were conducted after the coating of all substrates with poly(L-lysine) (PLL). For each condition parameters like cell adhesion, neurite number and length of the longest neurite were determined, as these are key when assessing the potential of a substrate to promote axonal regeneration. It is hypothesized that the observed differential cell behavior is related to the materials' nanomechanical properties that were characterized in this study. The involved cell signaling pathway was also investigated.

## Results

### 1.1 Cortical neurons adhere and extend neurites in a PLL dependent manner

**Figure 1:**
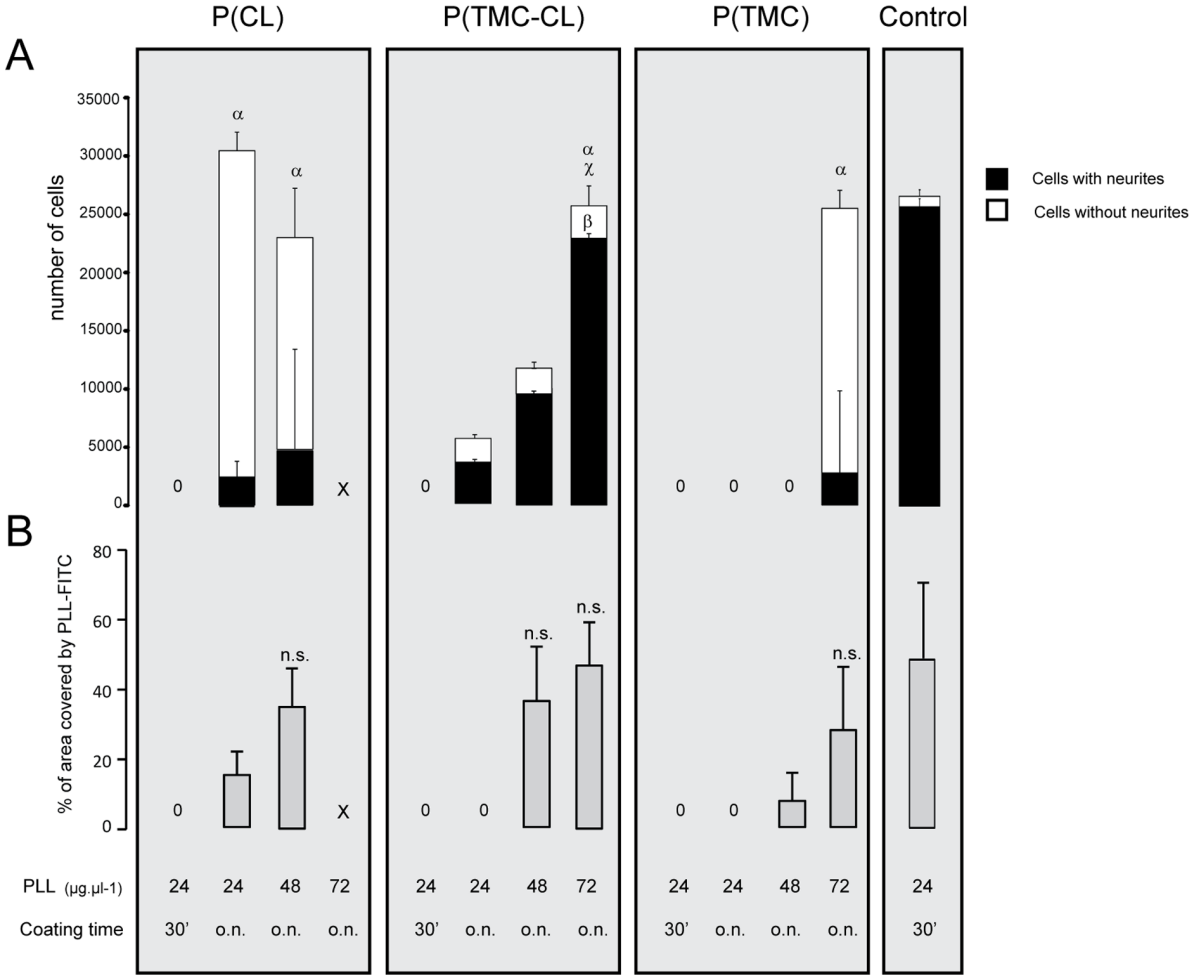
Cortical neuron culture on PLL coated films of P(TMC-CL) and respective homopolymers (2.7×10^4^ viable cells were seeded per sample). **A.** Number of cortical neurons with and without neurite extensions on polymeric surfaces coated with aqueous solutions at different concentrations of PLL. Glass coated with 24 µg.µl^−1^ of PLL for 30 minutes was used as control. (n = 3 independent studies, mean ± SD, p<0.05) **B.** Percentage of PLL covered surface area as a function of the coating conditions. (n = 3, mean ± SD, p<0.05). x = condition not tested, 0 = null value. n.s. = non-significantly different from the control, α = total number of cells not significantly different from the control, β = number of cells with neurite extensions not significantly different from the control and χ = number of cells without extensions not significantly different from the control.

As a first step in assessing P(CL), P(TMC-CL) and P(TMC) compatibility with the CNS and their potential application in devices for neuroregeneration, polymeric discs were tested as substrates for cortical neuron growth in vitro. Cortical neurons were seeded on PLL coated polymeric films and were found to adhere to the tested substrates in a PLL concentration dependent manner (Fig. 1A). Cell number and neurite outgrowth on the coated polymeric films were evaluated using coverglasses coated with a PLL concentration of 24 µg.µl^−1^ for 30 min as control. Cortical neurons adhered in comparable numbers to the control when the polymeric films were coated overnight with 24 µg.µl^−1^ and 48 µg.µl^−1^ of PLL in the case of P(CL) films, and 72 µg.µl^−1^ of PLL in the case of TMC containing films (see Fig. 1A). However, only on P(TMC-CL) the majority of adhered cells was able to extend neurites as in the control.

To explain this PLL-dependent behaviour, the amount of PLL adsorbed to the polymeric films surface was evaluated by fluorescence quantification of PLL-FITC coated samples (see fig. S1 for PLL-FITC coating fluorescence images). As one can observe in Fig. 1B, the surface area covered by PLL (in %) was only comparable to the control conditions when the polymeric films were treated with a PLL solution of at least 48 µg.µl^−1^ and 72 µg.µl^−1^ in the case of the CL containing materials and P(TMC), respectively. Consequently, cell adhesion can be correlated with the PLL adsorption profile to the polymeric films.

Taking into consideration the obtained results, both in terms of cell adhesion and neurite extension, the coating conditions used in the subsequent studies were established to be polymer surface treatment overnight with 48 µg.µl^−1^ for P(CL) and 72 µg.µl^−1^ for P(TMC-CL) and P(TMC).

### 1.2 P(TMC-CL) stimulates axonal elongation

**Figure 2:**
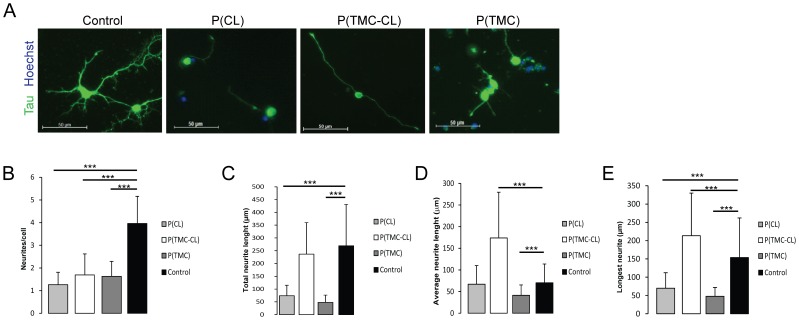
Effect of the PLL coated surfaces on neurite elongation and cellular polarization. **A**. Fluorescently labeled cortical neurons, immunostained for TAU (green); nuclei are counterstainned with Hoechst (blue); **B.** Number of primary neurites per cell; **C.** Total neurite length; **D.** Average neurite length and **E.** Length of the longest neurite. (n = 130 cells, mean ± SD, *** for p<0.001).

In order to evaluate neurite outgrowth on the different PLL-coated polymeric surfaces, the number of neurites per cell, as well as the neurite length were determined. As seen in Fig. 2A, neurons behave differently on each surface. More than 80% of the cells seeded on polymeric films show one or two neurites, while more than 80% of the cells seeded on glass (control) present between 3 to 5 neurites (Fig. 2B). Furthermore, as shown in Fig. 2A neurons seeded on the polymeric surfaces exhibit a lower degree of branching than those seeded on glass. However, on P(CL) and P(TMC) the adhered cells show smaller neurites than on P(TMC-CL) and the control (Fig. 2C–E). Despite the fact that for P(TMC-CL) the total neurite length was similar to the one observed on glass, given the lower number of neurites per cell in this condition, the average neurite length was higher (Fig. 2D). More remarkably, the length of the longest neurite was increased relatively to the control (Fig. 2E).

### 1.3 P(TMC-CL)'s nanomechanical properties

**Figure 3:**
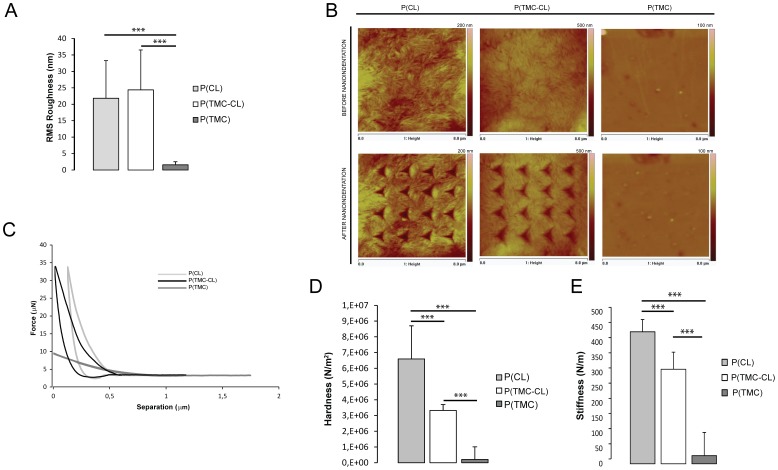
Morphology and mechanical properties of the tested polymeric surfaces. **A**. Root mean square (RMS) roughness of all polymeric surfaces; **B**. Representative photographs of the polymeric surfaces before and after nanoindentation; images are color coded, showing elevated areas in bright and lower areas in dark color. **C**. Representative nanoindentation force-displacement curves; D. Mean hardness values of all polymeric surfaces, calculated for the maximum load and E. Mean stiffness values for all polymeric surfaces. (n = 60 indentations, mean ± SD, *** for p<0.001).

As shown in Fig. 3A, atomic force microscopy (AFM) analysis indicated that the RMS roughness was similar for P(CL) and P(TMC-CL), with mean values of 21.8±11.5 nm and 24.4±12.1 nm respectively, while significantly lower for P(TMC) with a mean value of 1.6±1.0 nm.

Nanoindentation is one of the most versatile techniques and particularly suited for the measurement of localized mechanical properties on the surface of materials [26]. Representative photos of the nanoindentations and force/displacement curves are represented in Fig. 3B and C, respectively. These show that P(CL) has a greater resistance to deformation in relation to the other two materials tested, as the force needed to achieve the same displacement is higher than for P(TMC-CL) or P(TMC). As shown in Fig. 3 D–E stiffness and hardness values are significantly different between the three different substrates. A stiffness value of 312±56.4 N.m^−1^ and a hardness of 3.32×10^6^±0.373×10^6^ N.m^−2^ was found for P(TMC-CL), while P(CL) shows the highest values with a stiffness of 435±40.4 N.m^−1^ and a hardness value of 6.60×10^6^±2.11×10^6^ N.m^−2^. As seen in the photos before and after nanoindentation, P(TMC) samples recover almost completely from the indentations and, consequently, show stiffness and hardness values close to zero.

### 1.4 P(TMC-CL) promotes restraining of myelin inhibition

**Figure 4:**
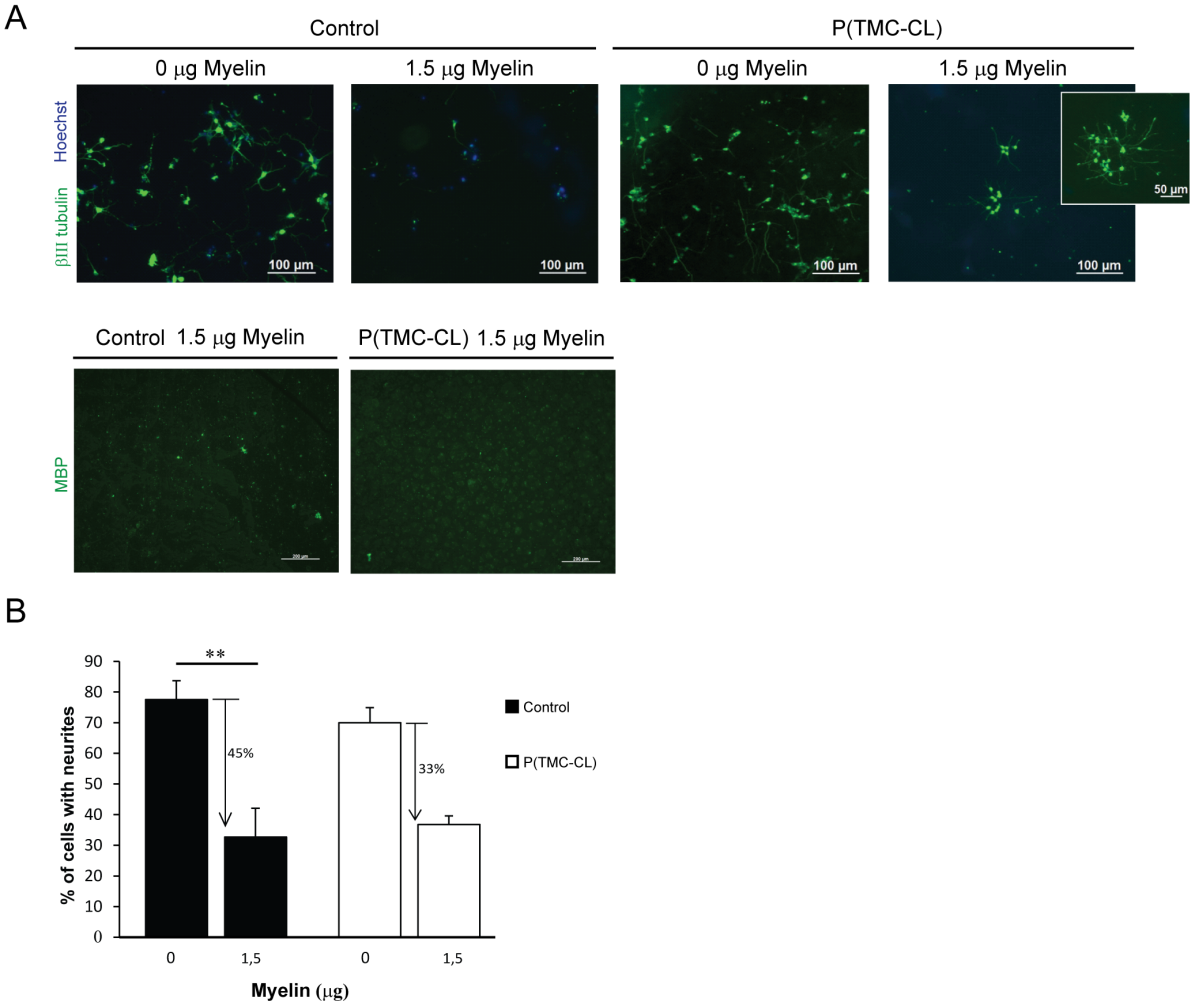
Effect of CNS myelin on neurite outgrowth of cortical neurons cultured for 4 days on PLL-P(TMC-CL) substrates coated with CNS myelin. **A.** Cortical neurons are immunostained for β-III tubulin (green) and nuclei are counterstainned with Hoechst (blue); myelin coating is immunostainned for MBP (green), surfaces were fully covered by myelin (see fig. S2 for myelin quantification) **B.** Effect of myelin on the ability of neurons to extend processes is presented as the % of cells with neurites in relation to the total number of cells. (n = 3 independent studies, mean ± SD; ** for p<0.01).

Myelin-associated inhibitors (MAIs) are present at a spinal cord lesion site and are known to be among the major impediments of the spontaneous axonal regeneration after SCI. Cortical neurons were seeded on myelin coated surfaces with a reduction of adherent cells of 50 and 55% for P(TMC-CL) and glass, respectively. P(TMC-CL) was chosen from the three tested surfaces as it showed the best results for neuronal adhesion and neurite extension, presenting a positive influence on axonal elongation. As seen in Fig. 4, when comparing surfaces coated and uncoated with myelin, the number of cells with neurites is smaller in the first case. Nevertheless, this decrease is not significant on P(TMC-CL) seeded neurons in contrast to the control where this reduction is statistically significant (p<0.01).

### 1.5 GSK3β signalling pathway mediates neuronal behaviour on P(TMC-CL) substrates

**Figure 5:**
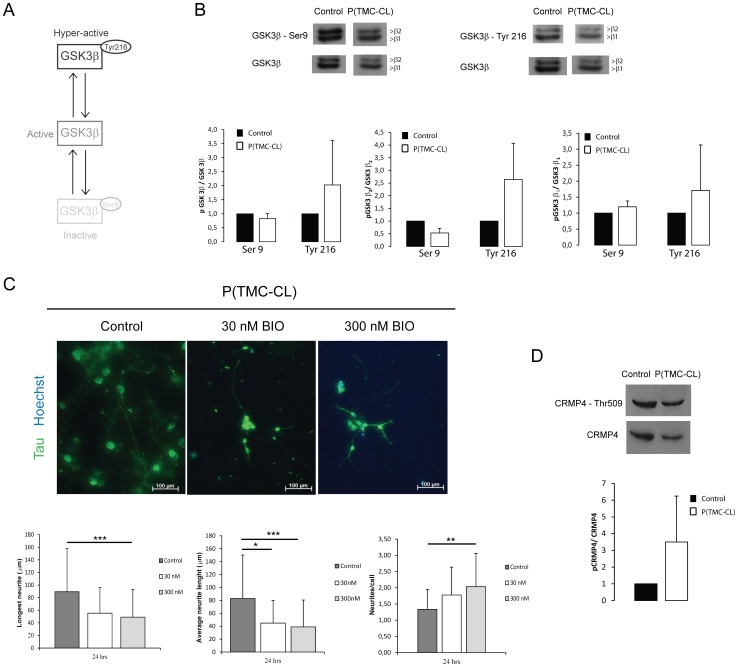
Analysis of GSK3β in cortical neurons plated on P(TMC-CL) and effects of GSK3β inhibition on neurite extension. **A.** Schematic representation of the different phosphorylation forms of GSK3β and their activity status; **B.** Analysis of the phosphorylated forms of GSK3β by western blot. Representative blots are shown. Expression levels of GSK3β isoforms, β1 and β2, are presented and quantified individually or together. (n = 3 independent studies, average ± SD); **C.** Morphology of neurons (immunostained for TAU in green and nuclei counterstained in blue) cultured for 24 hours in the presence of DMSO (control) or in the presence of 6-bromoindirubin-3′-acetoxime (BIO) at 30 and 300 nM. Quantifications of the longest neurite, average neurite length and the number of neurites per cell are shown (n = 130 cells, mean ± SD, * for p<0.05, ** for p<0.01 and *** for p<0.001); **D.** Determination of CRMP4 phosphorylation levels in cortical neurons plated for 4 days on control or P(TMC-CL). Representative western blot is shown and below the quantification (n = 3 independent studies, average ± SD).

Glycogen synthase kinase 3β (GSK3β) is implicated in many processes in the nervous system and is known to play a critical role in the regulation of neuron physiology. It is highly expressed in neurons and crucial for the establishment of neuronal polarity, as well as for the establishment of the branching-elongation equilibrium [27], [28], [29]. In view of this knowledge, the involvement of GSK3β as a mediator of P(TMC-CL) effect on neurite formation and axonal outgrowth was examined. GSK3β is regulated by phosphorylation and its activity can be reduced by phosphorylation at Ser-9. Contrarily, tyrosine phosphorylation at Tyr-216 increases the enzyme's activity (Fig. 5A) [30], [31]. As shown in Fig. 5B cortical neurons seeded on P(TMC-CL) present lower levels of GSK3β Ser-9 phosphorylation and higher level of Tyr-216 phosphorylation, in comparison to neurons cultured on glass. This indicates that neurons seeded on P(TMC-CL) display more kinase activity than those on glass. It is also perceptible from Fig. 5B that the GSK3β isoform that is differently expressed is GSK3β_2_, which is known to be expressed exclusively in the nervous system [31], [32]. To further confirm the involvement of GSK3β as a mediator of the P(TMC-CL) effect on axonal outgrowth and number of neurites per cell, cortical neurons were cultured in the presence of a GSK3 pharmacologic inhibitor - 6-bromoindirubin-3′-acetoxime (BIO). It is expected that inhibiting GSK3 activity should inhibit the polymeric surface's effect on cortical neurons. In fact, as shown in Fig. 5C, when BIO is added to the culture medium one can observe a decrease in the length of the longest neurite and in the average neurite length, as well as an increase on the number of neurites per cell. These effects occur in a dose-dependent manner, with the highest concentration of BIO tested (300 nM) leading to statistically significant differences. Alabed et al. [33] have established that GSK3β phosphorylation and consequent inactivation, regulates the interaction of CRMP4 and RhoA through CRMP4 de-phosphorylation. If this mechanism is active in our setup, phospho-CRMP4 levels should be higher for neurons seeded on P(TMC-CL). To test this hypothesis, the levels of CRMP4 phosphorylation in cortical neurons seeded on P(TMC-CL) and glass surfaces were assessed. As expected, phospho-CRMP4 levels were increased for neurons cultured on P(TMC-CL) as shown in Fig. 5D.

## Discussion

In the aftermath of a SCI, a glial scar is formed. Despite its key role in constraining the damaging effects caused by the lesion, the glial scar also prevents axon regeneration. The astroglial scar not only contains secreted and transmembrane molecular inhibitors of axon growth but also constitutes an almost impenetrable physical barrier to regeneration [4]. Consequently, it was hypothethized that by creating a favourable environment at the lesion site, one will be able to enhance axonal regeneration and ultimately promote some gain of function. Therefore, the use of an implantable scaffold to bypass the glial scar area is one of the promising approaches being investigated to promote spinal cord regeneration. A prerequisite in the design of such biomaterial is its biocompatibility, which in this context means that it must support neuronal survival and axonal growth. The aim of this study was, therefore, to investigate the suitability of P(CL), P(TMC-CL) and P(TMC) as substrates for spinal cord regeneration purposes.

One of the most commonly used strategies to assess neuronal behaviour in vitro when testing biomaterials for nerve regeneration applications is to evaluate axonal growth [33], [34], [35], [36], [37]. In the present work rat cortical neurons were firstly seeded on the PLL-coated polymeric substrates to assess adhesion and neurite outgrowth ability. PLL is a synthetic homo-poly (amino acid), characterized by an isopeptide bond between the ε-amino and the α-carboxyl groups of L-lysine, commonly used to coat cell culture substrates [38]. Initially, the polymer surface coating conditions were optimized - PLL concentration and time of contact - in order to achieve a comparable surface area covered by PLL and, consequently, similar cell adhesion in all tested surfaces. The observed PLL dependent behaviour can be explained by the different adsorption capacity of PLL on polymeric and glass surfaces. Differences that can be attributable to the surface properties of the tested materials, as these polymers present a more hydrophobic surface than glass [20]. Although after this process one could obtain comparable numbers of cortical neurons after 4 days of culture on the tested materials, significant morphological differences were found between neurons cultured on polymeric surfaces, particularly P(TMC-CL), and the control. Firstly, only on P(TMC-CL) the majority of neurons is able to extend neurites. Furthermore, our results show that among all the tested surfaces, including glass, seeding cortical neurons on P(TMC-CL) stimulates neuronal polarization and promotes axon elongation, as neurons on P(TMC-CL) show significantly enhanced neurite outgrowth and significantly lower numbers of neurites per cell. This switch to polarized and elongated morphology is noteworthy as successful regeneration requires that neurons survive and initiate rapid and directed neurite outgrowth [39], [40], [41]. A decreased number of neurites per cell were also found on P(CL) and P(TMC) but on these materials axonal outgrowth was significantly impaired. Moreover, while control neurons have, on average, twice the number of neurites of neurons seeded on P(TMC-CL), when one sums the length of all neurites of each cell (total neurite length) no significant differences are found. Altogether, cortical neurons seeded on P(TMC-CL) were found not only to be polarized but also to extend significantly longer neurites. To the best of our knowledge, no previous reports have shown this neuronal behaviour on any studied material.

The potential of materials to trigger specific cellular responses is getting to be a well established phenomenon mediated by a number of factors that range from the properties of the surface that contacts with the cell, to the mechanical properties of the material [16], [17], [42], [43], [44]. We have previously characterized the family of these copolymers and when varying the monomer ratio mainly the thermal and, consequently, the mechanical properties of these materials are drastically affected [21]. P(TMC) and P(TMC-CL) copolymers with high CL content are flexible and though materials that range from amorphous to semi-crystaline elastomers when the CL content increases. Therefore, here we hypothesise that surface topography and the nanomechanical properties of the tested materials play a key role in influencing cell behaviour. The local characterization of roughness, hardness and elastic properties of a wide range of materials has been reported including for thin films and biomolecules [45], [46], [47], [48] but so far the characterization of TMC-CL copolymers has not been performed. The roughness of the three tested polymeric surfaces was first investigated. Values of 22 nm and 24 nm were found for P(CL) and P(TMC-CL) respectively, while for the P(TMC) the roughness values were found to be significantly lower. In 2002, Fan et al. [49], [50] showed that neuronal cells adherence and survival is optimum on surfaces with a RMS roughness ranging from 10 to 50 nm. Taking this data in consideration, both P(CL) and P(TMC-CL) show not only similar but also optimum roughness values for neural adhesion and survival, while P(TMC) is outside this optimum roughness range. Therefore, the different neuronal behaviour on these surfaces cannot be explained simply by topography. Aiming to measure localized mechanical properties on the surface of the polymeric films, nanoindentations were performed and force-displacement curves obtained for each indentation. Mean hardness and stiffness values were calculated and significant differences were found between all polymeric surfaces, with P(TMC-CL) being significantly less resistant to deformation than P(CL) and significantly more resistant to deformation than P(TMC). Although roughness values were similar between P(CL) and P(TMC-CL) and within the optimum range, P(CL) was two times harder than P(TMC-CL), which could explain the different cellular behaviour on these surfaces, indicating that changes in stiffness and hardness values may have caused changes in cell morphology, specifically in axonal elongation.

Having observed the ability of P(TMC-CL) surfaces in promoting neuronal polarization and axonal elongation under normal cell culture conditions, the capacity of P(TMC-CL) to positively affect cortical neurons in a typical CNS inhibitory environment was tested, envisaging its application in the design of an axonal regeneration promoting strategy. While axons in the context of a mature mammalian CNS do not regenerate if damaged, the immature mammalian CNS is able to regenerate after injury [51], [52]. Probably the most notable difference between the mature and the immature nervous system is the presence of myelin [34]. Indeed, the limited regenerative capacity of the mammalian CNS is known to be partially due to myelin inhibition. So far, no biomaterial has shown the ability to restrain myelin inhibition unless blockers of myelin protein receptors were used [53]. Recently, Mohammad and co-workers have shown that a nano-textured self-assembled aligned collagen hydrogel was able to promote directional neurite guidance and restrain inhibition by a recombinant myelin-associated glycoprotein of dorsal root ganglia cultures [54]. To assess P(TMC-CL)'s neuronal effect under adverse, and more biologically relevant conditions, cortical neurons were seeded on P(TMC-CL) films in the presence of myelin. As expected, in the glass control surface we observed a statistically significant reduction of the number of cells extending neurites when cultured in the presence of myelin. In contrast, when P(TMC-CL) was used as a substrate, this reduction was not statistically significant (Fig. 4 B), suggesting that P(TMC-CL) is, to some extent, contributing to the promotion of the overcome of myelin inhibition. This is of extreme relevance as it has been already demonstrated that some degree of functional recovery can be obtained simply by counteracting the activity of myelin inhibition [55], [56]. The existence of a biomaterial that has the capacity to restrain this inhibition per se, without the need for the administration of antibodies or chemical inhibitors, can prove to be of great importance for therapeutic purposes.

The potential of materials to trigger specific cellular responses, such as interference and/or activation of defined pathways is extremely promising for tissue engineering. Stiffness and hardness sensing probably involves transduction into biological signals [15]. GSK3β is known to regulate axonal growth through the modification of the phosphorylation status of several microtubule-binding proteins and consequently the assembly of microtubules [31], [57]. Moreover, Alabed et al. [33] showed that the overexpression of active GSK3β attenuates MAI-dependent neurite outgrowth inhibition. For these reasons, GSK3 was studied as a possible mediator of P(TMC-CL)'s effect. Mammalian GSK3 is generated from two genes, GSK3α and GSK3β. GSK3 expression in neurons is further characterized by an alternative splicing of GSK3β originating two main variants: GSK3β_1_ and GSK3β_2_. GSK3β_2_ is specifically expressed in the nervous system [31]. GSK3β is regulated by phosphorylation and its activity is dependent on the balance between tyrosine (Tyr-216) and serine (Ser-9) phosphorylation as shown in Fig. 5A, with a reduction of activity if phosphorylated at Ser-9, and its increase if phosphorylated at Tyr-216 [30], [31]. Our results show that GSK3β is differently regulated in neurons seeded on glass and P(TMC-CL), with the latter showing lower levels of Ser9 phosphorylation, a site of GSK3β inactivation, and higher levels of Tyr216 phosphorylation, which facilitates the activity of GSK3β by promoting substrate accessibility [31]. Neurite elongation and neuronal polarization on P(TMC-CL) may be promoted by an increase GSK3β activity in vitro. The relationship between axonal elongation and GSK3β activity was further confirmed through pharmacological inhibition of GSK3 in vitro. As expected, inhibition of GSK3β blocked P(TMC-CL) effect, as there was a decrease in neurite length and an increase on the numbers of neurites per cell. Cells seeded on P(TMC-CL) and treated with BIO acquired a morphology that resembles more closely the neurons seeded on glass Fig. 2A.

Activation of GSK3β activity occurs in cortical neurons when these are cultured on P(TMC-CL), resulting in an increase in neurite outgrowth and decrease on the number of neurites per cell. Increased axonal outgrowth in the presence of higher GSK3β activity has also been shown in prior reports, for cerebellar, dorsal root ganglia and hippocampal neurons [28], [33], [58].

The Rho signalling pathway is known to play an important role in neuronal growth regulation and it has been shown that inhibitors of RhoA, and/or its downstream effector Rho kinase, facilitate growth on myelin substrates [59], [60]. Wozniak et al. [16] have studied the effects of stiffness on cell shape and shown that ROCK mediated contractility is essential for breast epithelial cells to sense the biophysical properties of the surrounding environment. Alabed et al. [61] have identified CRMP4 as a protein that functionally interacts with RhoA to mediate neurite outgrowth. Later on, this team has found that CRMP4-RhoA interaction is regulated by dephosphorylation of CRMP4 as a direct consequence of GSK3β inactivation by phosphorylation at Ser-9 [33]. This observation indicates that overexpression of GSK3β and consequent inhibition of CRMP4-RhoA complex formation may be protective in the context of myelin inhibition. Our findings are consistent with Alabed et al. [33] as for neurons seeded on P(TMC-CL), which show higher levels of GSK3β activity and longer neurites the levels of phospho-CRMP4 are higher than in glass seeded neurons. Overall these results suggest that the activation of GSK3β activity, and consequent neurite elongation, is mediated by the surface mechanical properties of P(TMC-CL).

## Conclusions

This work shows that P(TMC-CL) with a high CL content can promote axonal regeneration, prompting neurons into a regeneration mode, even under inhibitory conditions. This effect is mediated by the GSK3β signalling pathway, which is triggered by P(TMC-CL)'s surface mechanical properties.

P(TMC-CL) being a material that can been processable in a variety of shapes and forms, including porous conduits and electrospun fibers, it presents itself as a valuable tool in the design of new strategies for application in the treatment of spinal cord lesions, while supporting axonal growth and taming myelin dependent neurite outgrowth inhibition without the need of the administration of any therapeutic drug.

## Materials and Methods

### 4.1 Polymeric film preparation

Poly(trimethylene carbonate) (P(TMC), poly(ε-caprolactone) (P(CL)) and poly(trimethylene carbonate-co-ε-caprolactone) (P(TMC-CL)) with 11 mol % of TMC were synthesized as previously described [20]. Briefly, prior to polymerization ε-caprolactone monomer (Fluka) was dried overnight over CaH_2_ and distilled under reduced pressure. Trimethylene carbonate was obtained from Boehringer Ingelheim (Germany) and used as received. Polymerizations were conducted by ring-opening polymerization in an argon atmosphere using stannous octoate as a catalyst. All polymerizations were carried out for a period of 3 days at 130°C±2°C. The obtained polymers were purified by dissolution in chloroform and subsequent precipitation into a ten-fold volume of ethanol. The precipitated polymers were recovered, washed with fresh ethanol and dried under reduced pressure at room temperature (RT) until constant weight. The prepared polymers were characterized with respect to chemical composition by nuclear magnetic resonance (NMR). Four hundred MHz ^1^H-NMR (BRUKER AVANCE III 400) spectra were recorded using solutions of polymer in CDCl3 (Sigma). Number average and weight average molecular weights (Mn and Mw, respectively), polydispersity indexes (PDI) and intrinsic viscosities ([η]) of the (co)polymers were determined by gel permeation chromatography (GPC, GPCmax VE-2001, Viscotek, USA). The setup was equipped with ViscoGEL I-guard-0478, ViscoGEL I-MBHMW-3078, and ViscoGEL I-MBLMW-3078 columns placed in series and a TDA 302 Triple Detector Array with refractometer, viscometer, and light-scattering detectors, allowing the determination of absolute molecular weights. All measurements were performed at 30°C, using chloroform as the eluent at a flow rate of 1.0 ml.min^−1^. The obtained results are compiled in Table 1.

**Table 1:** Characteristics of the synthesized and purified P(TMC-CL) (co)polymers.

Polymer	TMC contenta) (mol %)	Mnb)	Mwb)	PDIb)	[η]b) (dl/g)
P(TMC)	100	2.16×10^5^	3.03×10^5^	1.4	4.02
P(TMC-CL)	11	0.37×10^5^	0.72×10^5^	1.9	1.46
P(CL)	0	0.28×10^5^	0.50×10^5^	1.8	1.04

a) Determined by 1H NMR on specimens purified by precipitation;

b) Determined by GPC at 30°C using chloroform as the eluent.

Polymer films of 250 µm in thickness were prepared by casting the polymer solution in chloroform onto glass Petri dishes. After drying the films under reduced pressure at RT, disks with a diameter of 14 mm were punched out. Prior to cell culture, disks were sterilized by two incubation steps in a 70% (v/v) ethanol solution for 15 min, followed by two rinsing steps of 15 min in autoclaved MilliQ water (Millipore). After sterilization, polymer disks were placed in 24-well tissue polystyrene plates (BD Biosciences) and fixed with autoclaved silicon o-rings (EPIDOR, Barcelona).

### 4.2 Cortical neuron cell culture

Prior to cell seeding the air side surface of the polymeric disks was coated with 200 µl of a poly(L-lysine) (PLL, Sigma) solution in a concentration ranging from 24 to 73 µg.µl^−1^, at 37°C for 30 minutes or overnight and, subsequently, rinsed with autoclaved MilliQ water. Coverglass (Menzel) coated with 24 µg.µl^−1^, at 37°C for 30 minutes was used as control.

Procedures involving animals and their care were conducted in compliance with institutional ethical guidelines and with the approval of Portuguese Veterinary Authorities – Direcção Geral de Veterinária (DGV); approval reference 0420/000/000/2007. Female wistar rats were housed in pairs with free access to food and water, under a 12-h light/12-h dark cycle. E17–E18 Wistar Han rat embryos were recovered by cesarean section of pregnant rats first anesthetized by intravenous injection of ketamine chlorohydrate (IMALGENE® 1000, Merail) and medetomidine hydrochloride (DOMITOR®, Pfizer Animal Health) to confirm pregnancy by palpation, and then euthanized with sodium pentobarbital 20% (EUTASIL, CEVA Sante Animal) by intravenous injection. The isolated cortices were dissociated for 30 min at 37°C in Hanks Balanced Salt Solution (HBSS) supplemented with 1.0 mM pyruvate, 2 mg.ml^−1^ albumin, and 10% (v/v) trypsin (all from Gibco). Viable cells (trypan blue exclusion assay) were seeded at a density of 2.2×10^4^ viable cells.cm^−2^ onto PLL-coated polymeric discs or coverglasses in 24-well cell culture plates. Neural cells were seeded in 300 µl of Dubelcco's Modified Eagle Medium (DMEM): Nutrient Mixture F-12 (F-12) (3∶1) supplemented with 10% (v/v) inactivated fetal calf serum (FCS) (all from Gibco). Two hours later, medium and o-ring were removed and 1 ml of Neurobasal medium supplemented with 0.5 mM L-glutamine, 2% (v/v) B27 supplement, 1% (v/v) Penicillin-Streptomycin and 0.5% (v/v) Gentamicin (all from Gibco) was added and polymeric discs turned upside down. Cultures were maintained at 37°C in a humidified atmosphere of 5% CO_2_. Culture purity was determined by immunocytochemistry as described further down. Half of the cell culture medium was changed on the third day of culture. After 4 days in culture, samples were treated for immunocytochemistry.

### 4.3 Poly(L-lysine) adsorption quantification

Polymeric disks were coated with PLL-FITC (fluorescein isothiocyanate) (Sigma) as described in the previous section. Coverglass coated with 24 µg.µl^−1^ of PLL, at 37°C for 30 minutes, was used as control. Polymeric discs coated with PLL-FITC were further mounted on microscope slides using an aqueous mounting media (Sigma) and observed with an inverted fluorescence microscope (Axiovert 200M, Zeiss, Germany). Image analysis was performed with ImageJ 1.44 software.

### 4.4 Atomic force microscopy

#### 4.4.1 Roughness analysis

The roughness of the polymer surfaces tested for cell culture was assessed by atomic force microscopy (AFM) using a PicoPlus scanning probe microscope interface with a PicoScan controller (Agilant Technologies, USA). A 10×10 µm^2^ piezoscanner was used in tapping mode, with a scan speed of 1 line.s^−1^. A bar shaped silicon cantilever (ACT probe, from AppNano), with a spring constant of 25–75 N.m^−1^ was used and roughness analysis was performed from scanned areas of 7×7 µm^2^ on five randomly chosen locations of each sample in air, at room temperature. The root-mean-square (RMS) roughness within the sampling area was determined using the WSxM scanning probe microscope software [62], according to
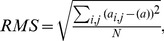
(1)where a represents the image height and N the total number of points.

#### 4.4.2 Nanoindentation

These measurements were performed at CEMUP (Centro de Materiais da Universidade do Porto), on a Veeco Metrology Multimode with Nanoscope IV controler (Veeco Instruments, Inc.) at RT conditions in Force-indent mode with a diamond tip, suitable for nanoindentation (DNISP Diamond-Tipped Probe from Veeco; spring constant 131 N.m^−1^). Deflection sensitivity of the cantilever was calibrated by indenting a sapphire surface. Nanoindentations were made for 1 second and the peak load was confined up to 30 µN for P(TMC-CL) and P(CL) and 6.5 µN for P(TMC). Force-displacement curves were obtained during loading and unloading for each indentation, and further used to determine hardness and stiffness values according to the Oliver and Pharr method [63]. For each polymeric substrate type, 60 indents were done on the film side tested for cell culture, covering 3 randomly chosen regions of 4 different samples per material. In each region, a set of 16 indents were made at a distance of 2 µm of each other. Stiffness was calculated as the slope of the tangent line to the unloading curve at the maximum loading point and hardness values were calculated for the maximum load and taking into consideration the shape of the indenter probe.

### 4.5 Neurite outgrowth on myelin coated polymer films

#### 4.5.1 Myelin isolation

Myelin was isolated from brains of C57BL/6 male mice, as previously described (for animal use ethics please see 5.2.) [64]. Briefly, the isolated brains were homogenized in 0.32 M sucrose and after centrifugation at 900 g, the post-nuclear supernatant was collected. The post-nuclear supernatant was carefully overlaid on an ultracentrifuge tube containing a 0.85 M sucrose solution on top of a 50% (w/v) sucrose cushion. After centrifugation for 1 hour at 37000 g at 4°C (Sorvall Pro80 centrifuge), the interphase between sucrose solutions was transferred to a new ultracentrifuge tube. Two rounds of osmotic shocks were performed by adding ice-cold water and centrifugation at 20000 g. The final myelin pellet was stored at −80°C until further use.

#### 4.5.2 Myelin Coating

The polymeric disks and glass control were first coated overnight with PLL as described above and washed with 0.1M NaHCO_3_. A myelin aqueous solution was subsequently dispensed onto the samples (total myelin protein 1.25 µg.cm^−2^), left to dry overnight in the laminar flow hood as previously described by Cai et al. [28], and further used as substrates for cortical neuron culture. Myelin coating of control and polymer surfaces was quantified by fluorescence microscopy after immune labeling of myelin with anti-MBP SMI94 (1∶500, Abcam).

### 4.6 Pharmacologic inhibition of glycogen synthase kinase 3

For neuronal outgrowth assays in the presence of a pharmacologic inhibitor of glycogen synthase kinase 3 (GSK3), a 30 or 300 nM solution of 6-bromoindirubin-3′-acetoxime (BIO) in dimethyl sulfoxide (DMSO) was added to cortical neuron cultures (DMSO final concentration 0.05% (v/v)) at two different time points: at seeding (t = 0) being in contact with cells for 4 days, and at the third day of culture (t = 3) being in contact with cells for 24 h. Neurons seeded on polymer discs in the presence of 0.05% (v/v) DMSO were used as controls. After 4 days in culture samples were treated for immunocytochemistry.

### 4.7 Immunocytochemistry

Cells were fixed for immunocytochemistry staining with 2% (v/v) paraformaldehyde at RT and further permeabilized and blocked in phosphate buffered saline (PBS) containing 5% (v/v) Normal Goat Serum (NGS) (Biosource) and 0.2% (v/v) Triton X-100 (Sigma). Primary antibodies were diluted in PBS containing 1% (v/v) NGS and 0.15% (v/v) Triton X-100, and incubated overnight in a humid chamber at 4°C. Secondary antibodies were applied for 1 h at RT and subsequently treated for nuclear counterstaining at RT with Hoechst (Molecular Probes) at 2 µl.ml^−1^. Samples were mounted directly in aqueous mounting medium and observed with an inverted fluorescence microscope.

Culture purity was ≥99% in cortical neurons as determined by mouse anti-glial fibrillary acid protein (GFAP) (1∶500, BD Biosciences)/mouse anti-vimentin (1∶100, Thermo Scientific)/mouse anti-oligodendrocyte marker 4 (O4) (1∶100, Chemicon)/rabbit anti-Tau (TAU protein) (1∶100, Sigma)/2 µg.ml-1 Hoechst fluorescent staining. Cells were counted from 18 radial fields and values were extrapolated to the total surface area of the sample (n = 3). For axonal outgrowth assessment the length of the longest neurite and total primary neurite outgrowth per cell were determined using AxioVision image analysis software. Neuronal processes were manually traced and quantified on 130 cells per condition. Three independent experiments were performed.

For neuronal outgrowth analysis on myelin inhibition studies, neurons were stained with anti-βIII tubulin (1∶500, Abcam) and myelin with anti-myelin basic protein (MBP) SMI-94 (1∶500, Abcam). The secondary antibodies used were anti-rabbit Alexa 488 (1∶500, Invitrogen), anti-mouse 594 (1∶1000, Invitrogen).

### 4.8 Western Blot

Cortical neuron lysates were prepared by washing cells with PBS and further lysed in buffer containing 20 mM 3-(n-morpholino)propanesulfonic acid (MOPS), 2 mM ethylene glycol tetraacetic acid (EGTA), 5 mM ethylenediaminetetraacetic acid (EDTA), 30 mM NaF, 60 mM β-glycerophosphate, 20 mM sodium pyrophosphate, 1 mM sodium orthovanadate, 1% (v/v) Triton X-100, 1% (v/v) DL-dithiothreitol (DTT), 1 mM phenylmethanesulfonyl fluoride (PMSF) and protease inhibitor cocktail (Amersham). Protein lysates (25–100 µg/lane) were run on a 12% SDS-PAGE gel and then transferred to a nitrocellulose membrane (Amersham). For Western analysis, membranes were blocked with blocking buffer (5% (wt/v) non-fat dried milk in tris-buffered saline (TBS) 0.1% (v/v) Tween 20) and incubated overnight at 4°C in 5% (wt/v) bovine serum albumin (BSA) in TBS 0.1% Tween 20 with primary antibodies. The following primary antibodies were used: rabbit anti-phospho-GSK3β Ser9 (1∶1000, Cell Signaling), rabbit anti-phospho-GSK3β Tyr216 (1∶2000, Santa Cruz Biotechnology), mouse anti-GSK3α/β (1∶2000, Santa Cruz Biotechnology), sheep anti-phospho- CRMP4 Thr 509 (1∶1000, Kinasource) and mouse anti-total CRMP4 (1∶500, Santa Cruz Biotechnology). After washing, membranes were incubated with secondary antibodies for 1 h at RT. The secondary antibodies used were anti-rabbit HRP (1∶10000, Jackson Immunoresearch), anti-mouse HRP (1∶10000, Thermo Scientific) and anti-goat/sheep (1∶10000, Binding Site). Proteins were detected using a chemiluminescent substrate Pierce ECL western blotting substrate (Thermo Scientific) according to the manufacturer's specifications. For each experiment representative western blots are shown. Phospho-protein expression was quantified by densitometry with QuantityOne software (BioRad) and levels were normalized to the total level of the same protein.

### 4.9 Statistical analysis

For statistical analysis, one-way ANOVA followed by Tukey's post-hoc test were used. When Gaussian distribution was not confirmed non-parametric test Man-Whitney was applied, using the Graphpad Prism program. Data is expressed as the mean ± standard deviation (SD) and p values of <0.05 were considered significant.

## Supporting Information

Figure S1 **Representative images of the PLL-FITC coating on the studied surfaces.**
(TIF)

Figure S2 **Distribution of the myelin coating.** **A)** Fluorescent quantification of the adsorbed myelin on P(TMC-CL) and glass surfaces.
(TIF)
