# Supplementary figures and images for: Poly(Trimethylene Carbonate-co-ε-Caprolactone) Promotes Axonal Growth
Source: PLoS One. 2014 Feb 27;9(2):e88593. doi: 10.1371/journal.pone.0088593 (PMC3937290; doi:10.1371/journal.pone.0088593)

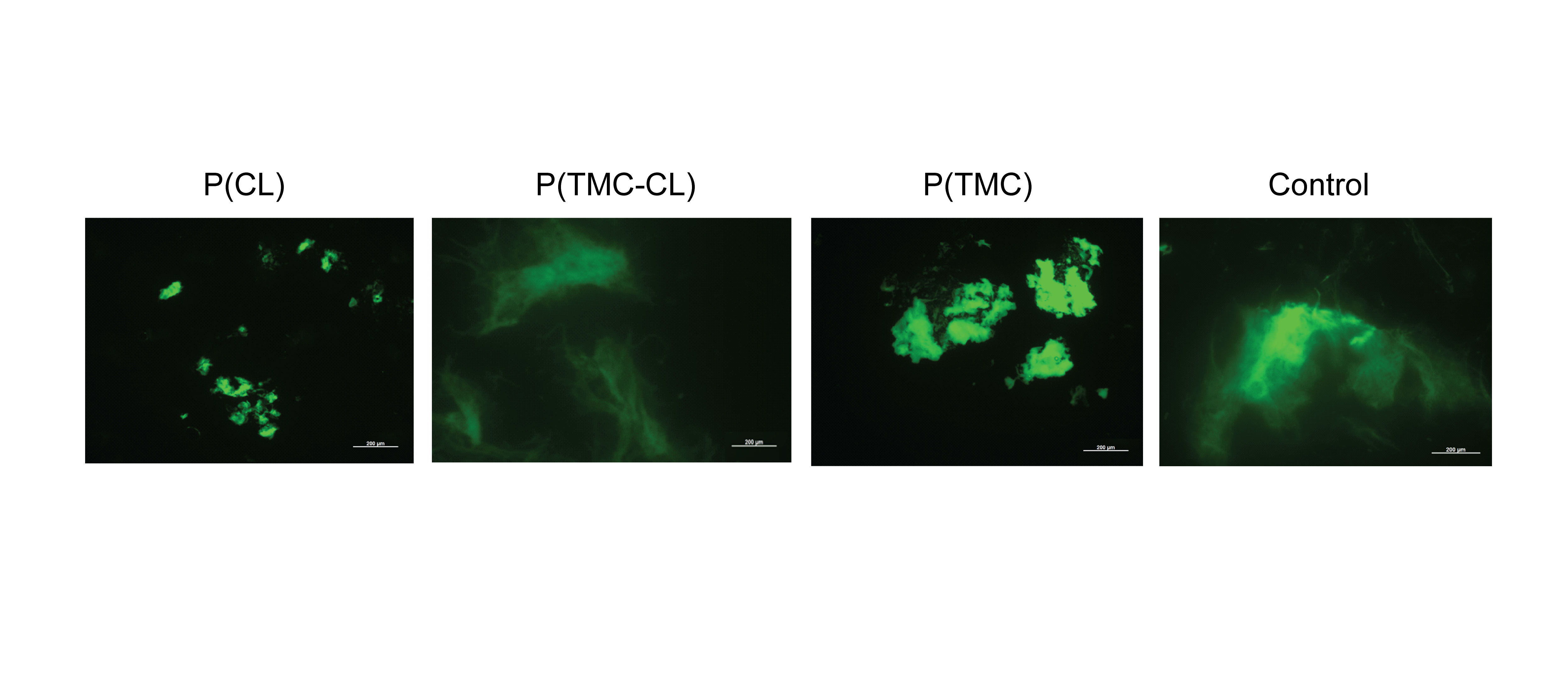

Supplement: Figure S1 — Representative images of the PLL-FITC coating on the studied surfaces. (TIF) [file pone.0088593.s001.tif]

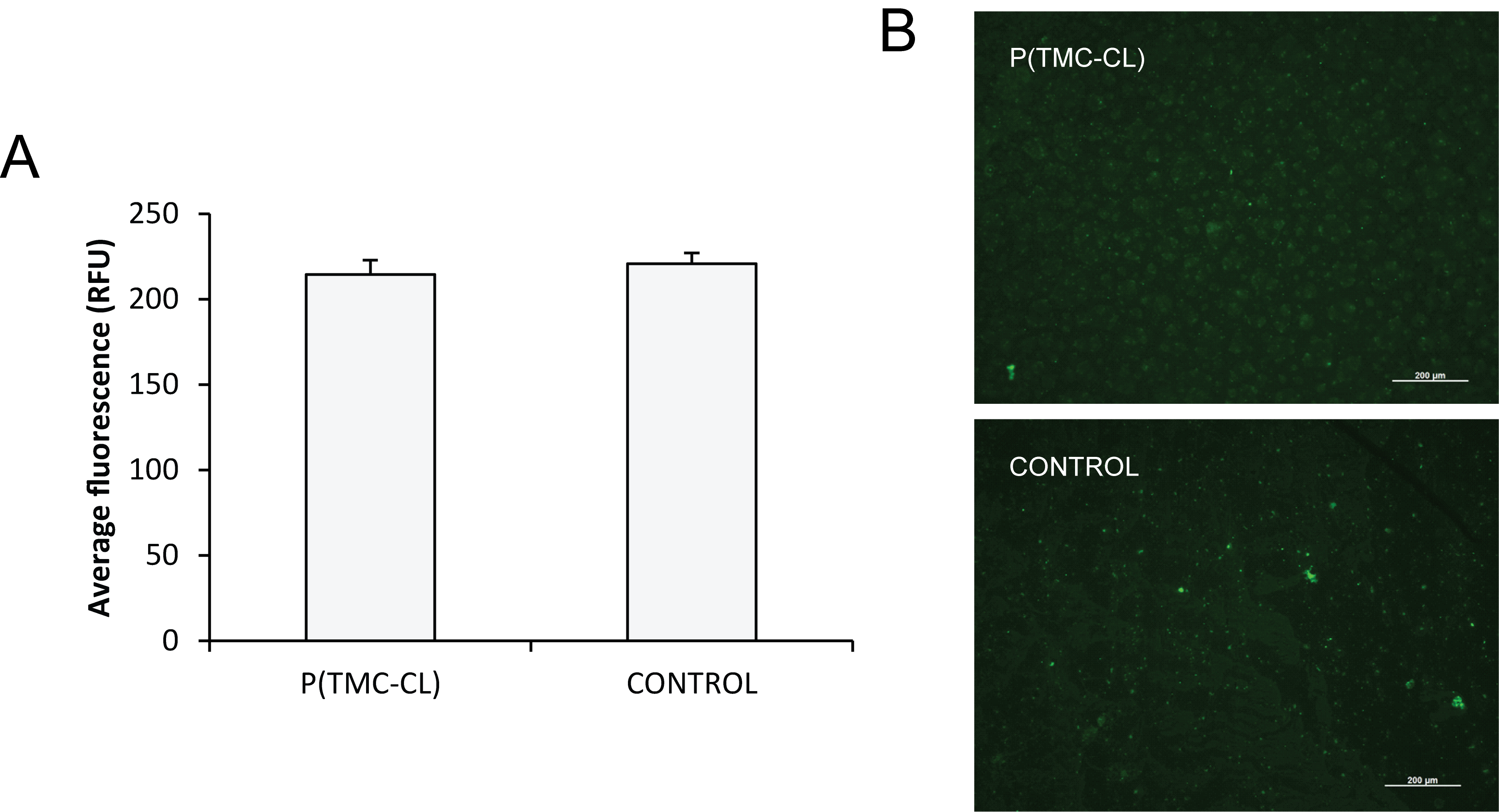

Supplement: Figure S2 — Distribution of the myelin coating. A) Fluorescent quantification of the adsorbed myelin on P(TMC-CL) and glass surfaces. (TIF) [file pone.0088593.s002.tif]
